# Red light regulation on growth, root-leaf architecture and photosynthetic performance of rice seedlings

**DOI:** 10.3389/fpls.2026.1919485

**Published:** 2026-09-09

**Authors:** Maofei Ren, Guiling Mao, Zhihao Wang, Jinping Ding, Junna Liu, Lingling Zhao, Xiangyan Wang, Boyan Du, Lijun Zhao, Hongxing Zheng, Chenyao Song, Qiankun Ding, Yanhao Ma

**Affiliations:** 1 Henan Environmental and Health Engineering Research Center, Henan Agricultural Remote Sensing Big Data Development and Innovation Laboratory & Henan Green Technology Innovation Demonstration Base, Shangqiu Normal University, Shangqiu, China; 2 College of Horticulture, Hunan Agricultural University, Changsha, China; 3 Henan Fengcai Agricultural Development Co. LTD, Suixian, China

**Keywords:** leaf-root structure, morphogenesis, photosynthesis, red light, rice

## Abstract

Given that the problem of insufficient light intensity is prevalent in the cultivation of rice seedlings under facility conditions, and light quality plays a crucial role in regulating crop growth, it is necessary to explore the most suitable proportion of red light for improving the quality and photosynthetic capacity of the seedlings. In this study, two different types of rice varieties (conventional rice, Xiangzaoxian 24, XZX24 and hybrid rice, Huazheyou 261, HZY261) were selected as experimental materials. A systematic investigation was conducted to examine the effects of different red light ratios on the morphological development of rice seedlings, the structure development of leaves and roots, as well as the photosynthetic characteristics. Six light quality treatments were applied, including white light (W, control), monochromatic red light (R), and combined white plus red light with different ratios (WR20, WR40, WR60, WR80). The results demonstrated that appropriate compound white-red light significantly coordinated shoot and root development; under the WR40 treatment (60% white light + 40% red light), the total seedling dry weight of the two varieties increased by 25.75% and 28.56% relative to the white light control, respectively. Gradually elevated red light proportion continuously boosted leaf pigment accumulation, with WR80 achieving the maximum total chlorophyll content, while WR60 optimized the non-photochemical quenching (NPQ) photoprotective capacity of leaves. Moderate red supplementation (WR20–WR60) maintained high Rubisco activity and upregulated the transcription of *OsRBCS2* and *OsRBCS3*, whereas the expression of *OsRBCS4* showed no significant difference across all light treatments. Single monochromatic red light failed to balance root branching and carbon assimilation efficiency despite promoting vertical leaf and stem elongation. This work provides quantitative spectral reference parameters for intelligent supplementary lighting in factory rice seedling raising, and clarifies the multi-dimensional physiological and molecular response differences of rice seedlings to graded red-white composite spectra. Further field verification trials are required to validate the applicability of the WR40 scheme under fluctuating natural environmental conditions.

## 1 Introduction

Rice (*Oryza sativa* L.) is the main food crop for more than half of the global population (An et al., 2026). The middle and lower reaches of the Yangtze River are the core area for double-cropping rice cultivation in China, fulfilling an indispensable strategic mission of ensuring national food security. Intensive factory-based seedling cultivation and mechanical transplanting have become the mainstream technical path to address the severe seasonal contradiction during the double-cropping rice planting period (Liu et al., 2025), effectively reducing the field occupation area, lowering labor input, and achieving standardized cultivation of vigorous seedlings, resulting in robust stems and well-developed root systems (Yang et al., 2026; Wang et al., 2017). Light is a unique limiting environmental factor in facility seedling cultivation, and light quality determines the entire process of rice light morphogenesis, the carbon assimilation process in photosynthesis, and the formation of stress resistance (Yu et al., 2020; Rashidi et al., 2026). With the rapid popularization of LED intelligent supplementary lighting technology in modern plant seedling cultivation factories (In et al., 2022; Ali et al., 2024; Zhang et al., 2024b), exploring the quantitative regulation laws of spectral components on seedling growth has become a hot research direction in facility crop cultivation and photophysiology, providing theoretical support for achieving low-cost, high-efficiency and precise control of the lighting environment in industrial seedling cultivation.

Due to the absorption and reflection of light by the transparent covering materials of the seedling cultivation facilities, the shading effect of the frame structure, and the attenuation of light by multiple layers of seedling trays, the spectral distribution and total radiation in greenhouses and vertical seedling racks differ significantly from the natural outdoor white light. Over the past decade, continuous meteorological monitoring data have shown that the annual sunshine duration and effective photosynthetic radiation in the Yangtze River rice-growing areas have continued to decline, while extreme continuous rainy weather frequently occurs in early spring during the seedling stage (Ren et al., 2023a; Fang et al., 2023). Under facility cultivation conditions, early rice seedlings often face multiple stresses, such as weak light, low temperature, and late spring cold damage, resulting in excessive growth, excessive elongation of stem nodes, poor root development, and reduced transplanting-induced tillering ability (Zhang et al., 2023; Wang et al., 2022, 2023). Conventional supplementary lighting systems commonly adopt incandescent lamps, high-pressure sodium lamps and metal halide lamps, which merely improve light intensity without optimizing spectral composition for crops. Therefore, they cannot fundamentally alleviate the physiological inhibition caused by weak light stress. Red light and blue light are the main spectral bands absorbed by rice leaves, and they are the core spectral bands that drive carbon assimilation in photosynthesis and the morphological development of seedlings (Chen et al., 2026; Anum et al., 2026; Borbély et al., 2022; Manodya et al., 2026). Therefore, in the weak light conditions within facilities, developing targeted red light gradient matching technology based on the natural white light background has become an urgent technical requirement for improving seedling quality.

Light quality exerts dual regulatory effects on energy supply and signal transduction. Rice senses red light signals through photoreceptor proteins and activates downstream transcriptional cascades, further regulating cell elongation, leaf expansion, and the transcription of Rubisco subunit family genes, thereby enhancing carbon assimilation efficiency (Lee et al., 2021; Cioć et al., 2022; Yu et al., 2019; Mao and Guo, 2018). Existing studies have shown that appropriate supplementation of red light can promote biomass accumulation in rice seedlings, increase photosynthetic pigment concentration, and alleviate damage caused by weak light stress to seedlings (Frąszczak and Kula-Maximenko, 2022; Ren et al., 2023a). However, most previous studies have three obvious limitations: first, most experiments used pure monochromatic red or blue light treatments, without considering natural white light as the basic light source, which cannot simulate the actual light environment of facility seedling cultivation rooms; second, only a few studies systematically compared the differences in responses of conventional rice and hybrid rice to different red light gradients. Third, the intrinsic relationship between the continuous proportion of red light, root-leaf structure differentiation, chlorophyll fluorescence characteristics, and photosynthesis gene expression has not been fully clarified, and there is a lack of multi-dimensional phenotypic, physiological, and molecular comprehensive evidence to formulate quantitative supplementary light standards. To fill these research gaps, systematic experiments with progressively graded white-red light ratios (red light proportion increased by 20% per treatment) were carried out to clarify the dose-effect response mechanism of rice seedlings to white-red composite spectra.

This study selected two representative rice varieties widely used in double-cropping rice production—Xiangzaoxian 24 (XZX24, conventional rice) and Huazheyou 261 (HZY261, hybrid rice) as experimental materials. Six light quality treatments were set up, including white light (W, control), monochromatic red light (R), and four groups of mixed white light and red light with different gradient ratios (WR20, WR40, WR60, WR80). Comprehensive measurements were conducted on the morphological characteristics of seedlings, root and leaf structure parameters, photosynthetic pigment content, chlorophyll fluorescence, gas exchange parameters, Rubisco activity, and the relative expression levels of three *OsRBCS* family genes. The core objectives of this study are to: (1) reveal the growth response patterns of rice seedling shoots and roots to varying red light proportions under white light background; (2) clarify the regulatory effects of red light ratios on photosynthetic capacity and photosynthesis-related gene expression from physiological and molecular perspectives; (3) identify the optimal white-red spectral combination for cultivating robust seedlings of the two rice cultivars. By optimizing red light allocation within composite spectra, this work provides theoretical support for crop light environment regulation and quantitative spectral references for intelligent supplementary lighting in factory rice seedling raising, while laying a basis for subsequent research on red light signaling pathways and the molecular breeding of shade-tolerant rice.

## 2 Materials and methods

### 2.1 Description of the experiment

This study utilized rice (Oryza sativa L.), including a conventional rice variety (Xiangzaoxian 24, XZX24) and a hybrid rice variety (Huazheyou 261, HZY261). The seeds of XZX24 and HZY261 were provided by Hunan Jinsenongyfeng Seed Industry Co., Ltd. and Hunan Jinjian Seed Technology Co., Ltd., respectively. All germinated rice seeds were sown and cultivated on rice-specific seedling growing substrate manufactured by Guangzhou Shengsheng Agriculture Co., Ltd (Execution standard: Q/SSNY07-2021). Uniform plug trays with dimensions of 54 cm (length) × 28 cm (width) containing 106 holes per tray were adopted, with 3 sprouted seeds sown into each hole (hole specifications: bottom diameter 10 mm, top diameter 17 mm, height 20 mm). Throughout the seedling raising cycle, seedlings were irrigated with tap water only, and no extra nutrient solution was supplied. Light quality treatments were initiated once the seedling emergence rate reached 95%. Before the light treatment, there was a dark treatment.

**Figure 1:**
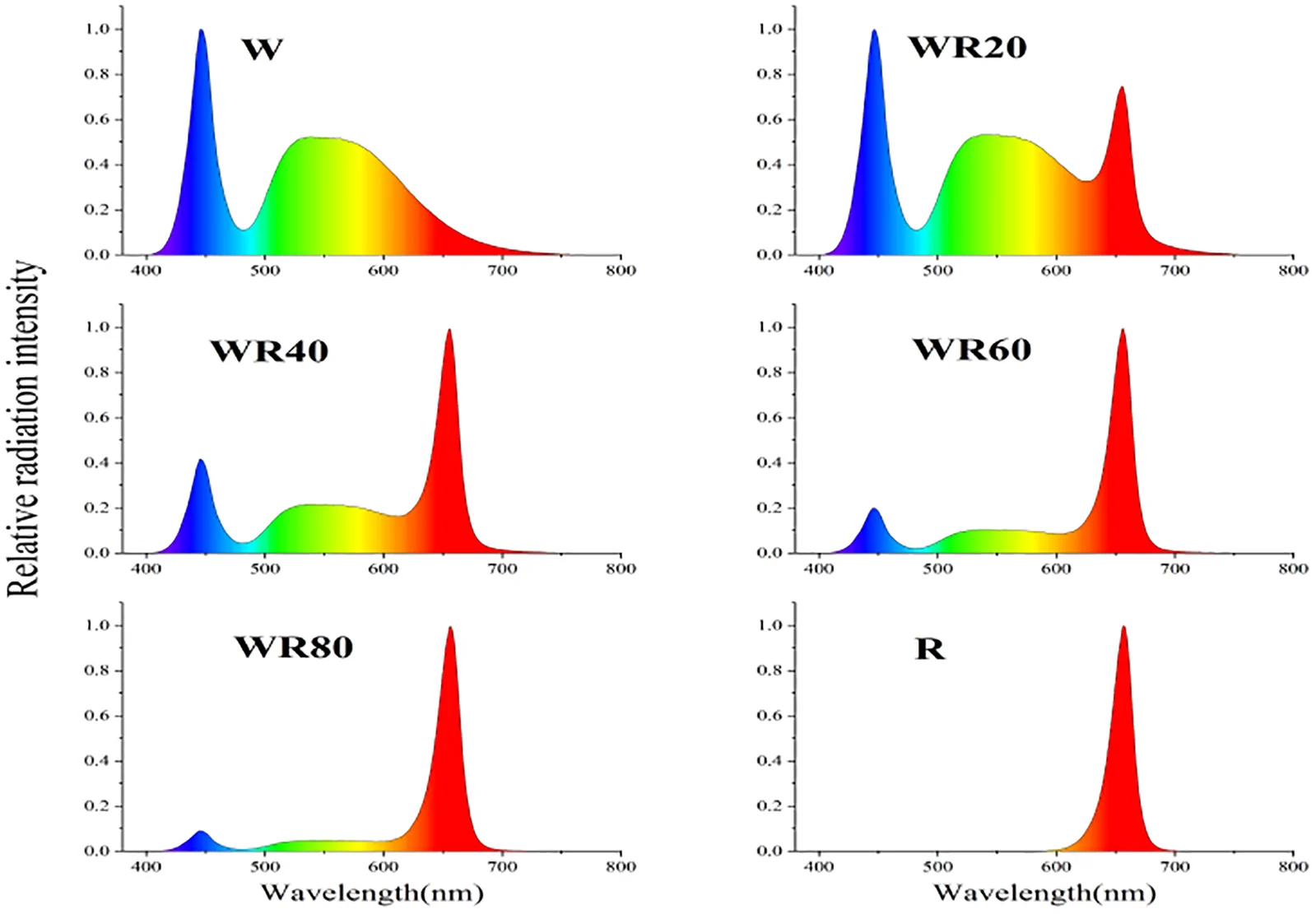
Illustrates the comparative spectral values across six treatment conditions. W: white light, WR20: 80% white light + 20% red light, WR40: 60% white light + 40% red light, WR60: 40% white light + 60% red light, WR80: 20% white light + 80% red light, R: 100% red light.

The experiment adopted a completely randomized design. Each light treatment contained 106 seedlings, with three independent biological replicates set for all six light quality groups. All seedling materials were evenly placed in an artificial climate chamber and subjected to six distinct light quality treatments. The growth conditions for the six light treatment scenarios were outlined as follows: (1) W: white light, (2) WR20: 80% white light + 20% red light, (3) WR40: 60% white light + 40% red light, (4) WR60: 40% white light + 60% red light, (5) WR80: 20% white light + 80% red light, (6) R: 100% red light. Daytime: light intensity = 100 ± 2 μmol·m^−2^·s^−1^, temperature = 25 ± 0.5 °C, average relative humidity = 60 ± 5%, and photoperiod = 12 h. Night: temperature = 15 ± 0.5 °C, average relative humidity = 80 ± 5%. The principal wavelength characteristic of red light is 665 nm. **Figure 1** displays the spectral data for the six processing techniques.

### 2.2 Morphological indicators

Following 28 days of continuous light treatment (the standard transplanting stage for mechanically raised rice seedlings), measurements were taken of seedling height, the size of the first-formed leaf sheath, and the stem base width. Concurrently, the LA-S analysis system (developed by Hangzhou Wanforest Inspection Technology Co., Ltd., Hangzhou City, China) was employed to assess the root systems and foliage of young rice plants, yielding pertinent data on both. Moreover, 60 plants were randomly selected from each replicate group and the mass values for fresh and dried specimens of every part (root/stem/leaf) of the young rice seedlings were determined. To determine the dry weight, the sampled tissue was first dried at 105 °C for 15 minutes, then placed in a 70 °C oven for drying until a constant weight was achieved, and finally weighed.

### 2.3 Pigment content

After 21 days of continuous light treatment, leaf pigment content was measured. Fresh rice seedling leaves were rinsed with sterile distilled water, and 0.1 g of cut leaf tissue was immersed in 10 mL of 95% anhydrous ethanol, followed by dark extraction for 12 h (Wang et al., 2026). The absorbance of the extract was measured at 665 nm (A_665_) and 649 nm (A_649_) using a UV–vis spectrophotometer (Shimazu, Tokyo, Japan), with a blank control of pure 95% anhydrous ethanol. The chlorophyll concentration calculation formulas referenced Wang et al. (2026):


$$\text{Chlorophyll a content }\left(\text{mg}\cdot {\text{g}}^{-1}\right)\;=\;13.95\;\times \;{\text{A}}_{665}\;-\;6.88\;\times \;{\text{A}}_{649}$$

$$\text{Chlorophyll b content }\left(\text{mg}\cdot {\text{g}}^{-1}\right)\;=\;24.96\;\times \;{\text{A}}_{649}\;-\;7.32\;\times \;{\text{A}}_{665}$$

### 2.4 Chlorophyll fluorescence

After a 21-day cultivation period, the chlorophyll fluorescence of rice seedlings grown under different light wavelengths was measured using the FluorPen 110/D (PSI, Czech Drahomorov). Before the measurement, the rice leaves were dark-treated for 20 minutes. For NPQ and ΦPSII determination, seedlings were pre-illuminated for 6 h under actinic light at 100 μmol·m^-2^·s^-1^. For the calculation of parameters such as NPQ, qP, Fv/Fm and ΦPSII, please refer to the research by Ren et al. (2023b).

### 2.5 Photosynthetic parameter analysis

Following 21 days of rice seedling cultivation under varied light conditions, the photosynthetic parameters of the rice seedlings were analyzed. An infrared gas analyzer was employed to assess the genuine photosynthetic performance (Pn), stomatal conductance (Gs), intercellular CO_2_ concentration (Ci), and transpiration rate (E) of rice seedlings (LI-6800, Li-COR, Lincoln, Oregon, USA) (Ren et al., 2023b).

### 2.6 Rubisco activity and gene expression

We measured the activity of Rubisco in the leaves under different light quality conditions, as well as the expression levels of genes related to Rubisco. The detailed information of specific primers and reference genes can be found in **Table 1**.

**Table 1:** The primer sequences of the Rubisco-related genes and OsActin.

Gene	Accession No.	Forward primer (5′-3′)	Reverse primer (5′-3′)
*OsActin*	Os03g0718100	CCACTATGTTCCCTGGCATT	GTACTCAGCCTTGGCAATCC
*OsRBCS2*	AK121444	CAGCAAGGTCGGATTCGTCT	CACACGAAACAAGGTGGGAG
*OsRBCS3*	AK068555	TTCCAAGGGCACAAGTCCAC	TAGGCAGCAATCCACCAGC
*OsRBCS4*	AK068266	GTGGATGAGTAAAGGGACAA	CAGGAGTAAATCAAGAGCGT

The primer synthesis was carried out by the company (Beijing Qingke Biotechnology Co., Ltd.). After 21 days of light treatment, 0.1 g of the second leaf middle part of the rice seedlings was collected and treated with liquid nitrogen. According to the manufacturer’s instructions, the total RNA of the sample was extracted using the RNA isolater Total RNA Extraction Reagent (Nanjing No. 5 Zonogen Biotechnology Co., Ltd.). Then, cDNA was synthesized using the reverse transcription kit. When analyzing the gene expression by real-time fluorescence quantitative instrument (CFX96 Touch, BIO-RAD America), the relative expression level of the sample was calculated as 2^-△△Ct^. All treatments were measured three times. For the determination methods and calculations, please refer to Ren’s description (Ren et al., 2023b).

### 2.7 Statistical analysis

All statistical plotting was conducted using Origin 2018 software. One-way analysis of variance (one-way ANOVA) was performed to evaluate the effects of different red light ratios on morphological, photosynthetic and molecular indicators of rice seedlings within each cultivar. After conducting a one-way analysis of variance, *post-hoc* multiple comparisons were performed using the least significant difference (LSD) test to separate the significant differences among different light treatment groups. All statistical calculations were completed with IBM SPSS Statistics 20 (IBM Corporation, Chicago, IL, USA).

## 3 Results

### 3.1 Plant height, first leaf sheath length, stem width, and leaf number

**Figure 2:**
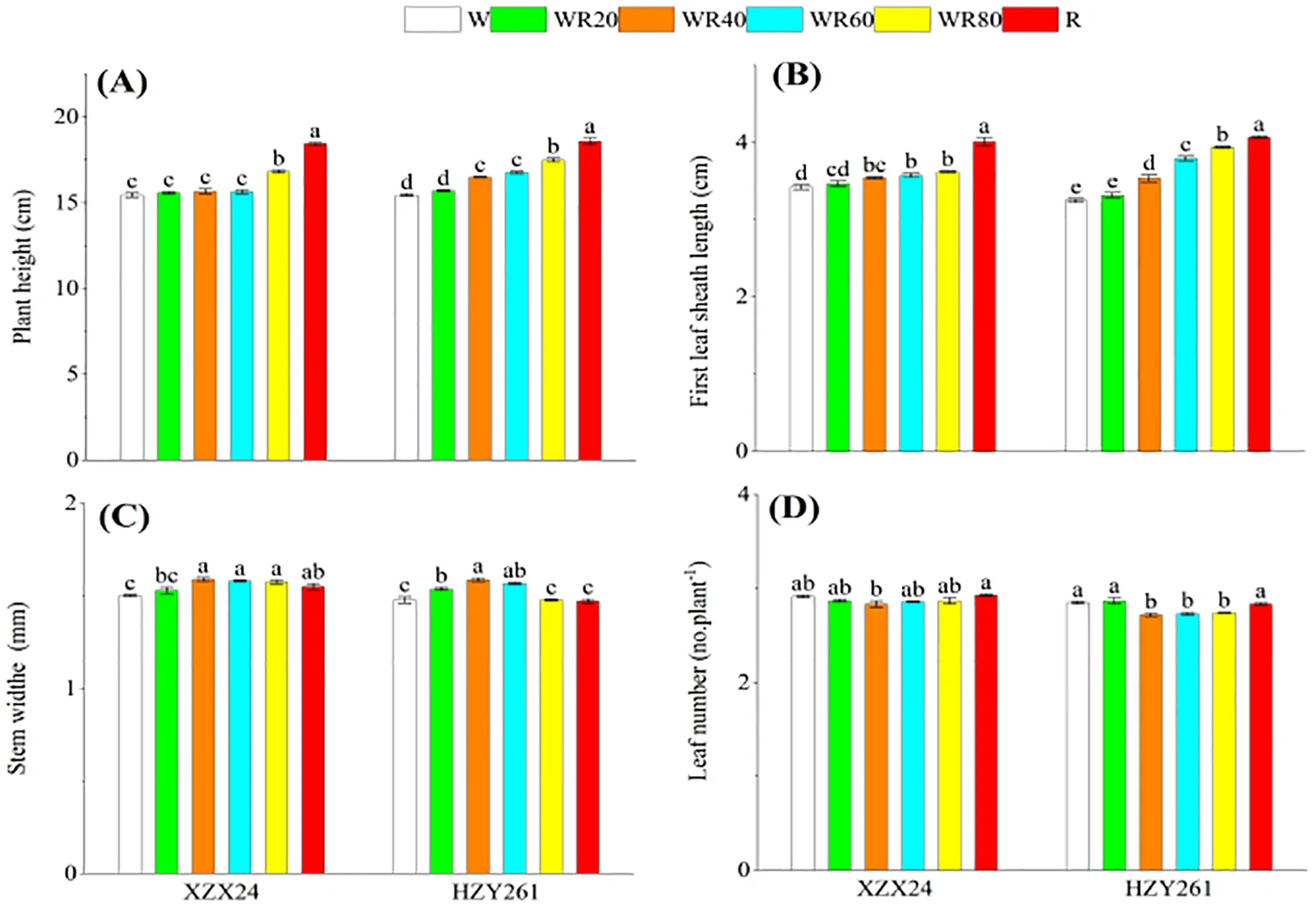
The influence of varying red light treatment ratios on the morphological characteristics of rice seedlings. **(A)** Plant height, **(B)** First leaf sheath length, **(C)** Stem width, **(D)** Leaf number, XZX24: Xiangzaoxian24, HZY261: Huazheyou261, W: white light, WR20: 80% white light + 20% red light, WR40: 60% white light + 40% red light, WR60: 40% white light + 60% red light, WR80: 20% white light + 80% red light, R: 100% red light. Based on the LSD test outcomes, distinct lowercase letters denote significant differences among various light quality treatments applied to the same rice variety, the differences are statistically significant at the 5% significance level. The error bars above the average values represent the standard error (n = 18).

Impacts on different red light treatment ratios on morphological attributes of rice seedlings, encompassing plant height, first leaf sheath length, stem width, and leaf number are shown in **Figure 2**. As the proportion of red light increases, the plant height and the length of the first leaf sheath of the rice seedlings show an upward trend, and reach the peak under the R treatment (**Figures 2A, B**). Compared with the W treatment, under the R treatment, the plant height of the XZX24 (HZY261) seedlings increased by 19.34%, and the first leaf sheath length increased by 17.19% (20.38% and 25.18% respectively) (**Figures 2A, B**). With the increase of the proportion of red light, the stem width of the rice seedlings first increases and then decreases. Compared with the W treatment, the stem width of the XZX24 (HZY261) seedlings treated with WR40 increased by 5.84% (7.35%) (**Figure 2C**).

### 3.2 Leaf growth

**Figure 3:**
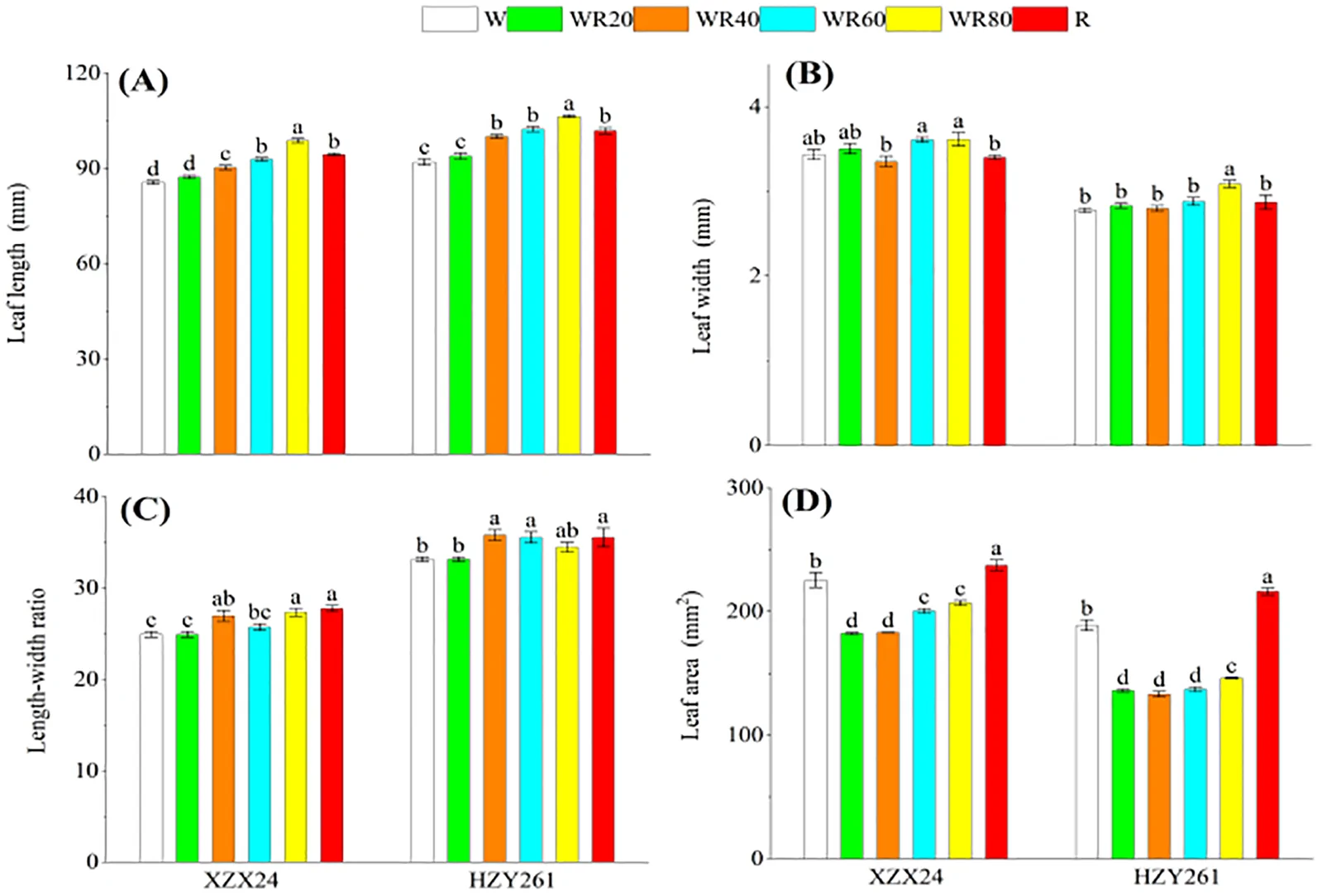
Impact of varying red light treatment proportions on developmental characteristics of rice seedling leaves: **(A)** Leaf length, **(B)** Leaf width, **(C)** length-width ratio, **(D)** Leaf area, XZX24: Xiangzaoxian24, HZY261: Huazheyou261, W: white light, WR20: 80% white light + 20% red light, WR40: 60% white light + 40% red light, WR60: 40% white light + 60% red light, WR80: 20% white light + 80% red light, R: 100% red light. Based on the LSD test outcomes, distinct lowercase letters denote significant differences among various light wavelength intervention methods applied to the same rice variety, the differences are statistically significant at the 5% significance level. The error bars above the average values represent the standard error (n = 18).

**Figure 3** illustrates how varying red light ratios influence the growth of rice seedling leaves. With a rise amid the red light percentage, rice seedlings leaf length increase, and reach the maximum value at the WR80 treatment (**Figure 3A**). Compared with the W treatment, the leaf length and leaf width of XZX24 (HZY261) seedlings under the WR80 treatment increased by 15.38% and 5.26% (15.63% and 11.23%), respectively (**Figures 3A, B**). The increase in red light ratio has an impact on the aspect ratio (length-width ratio) of rice seedlings. The leaf area of XZX24 rice seedlings (HZY261) seedlings reaches its peak under the R treatment. Compared with the W treatment, under treatment R, the leaf area of XZX24 (HZY261) seedlings showed a 5.29% (or 14.45%) increase (**Figure 3D**).

### 3.3 Root growth

**Figure 4:**
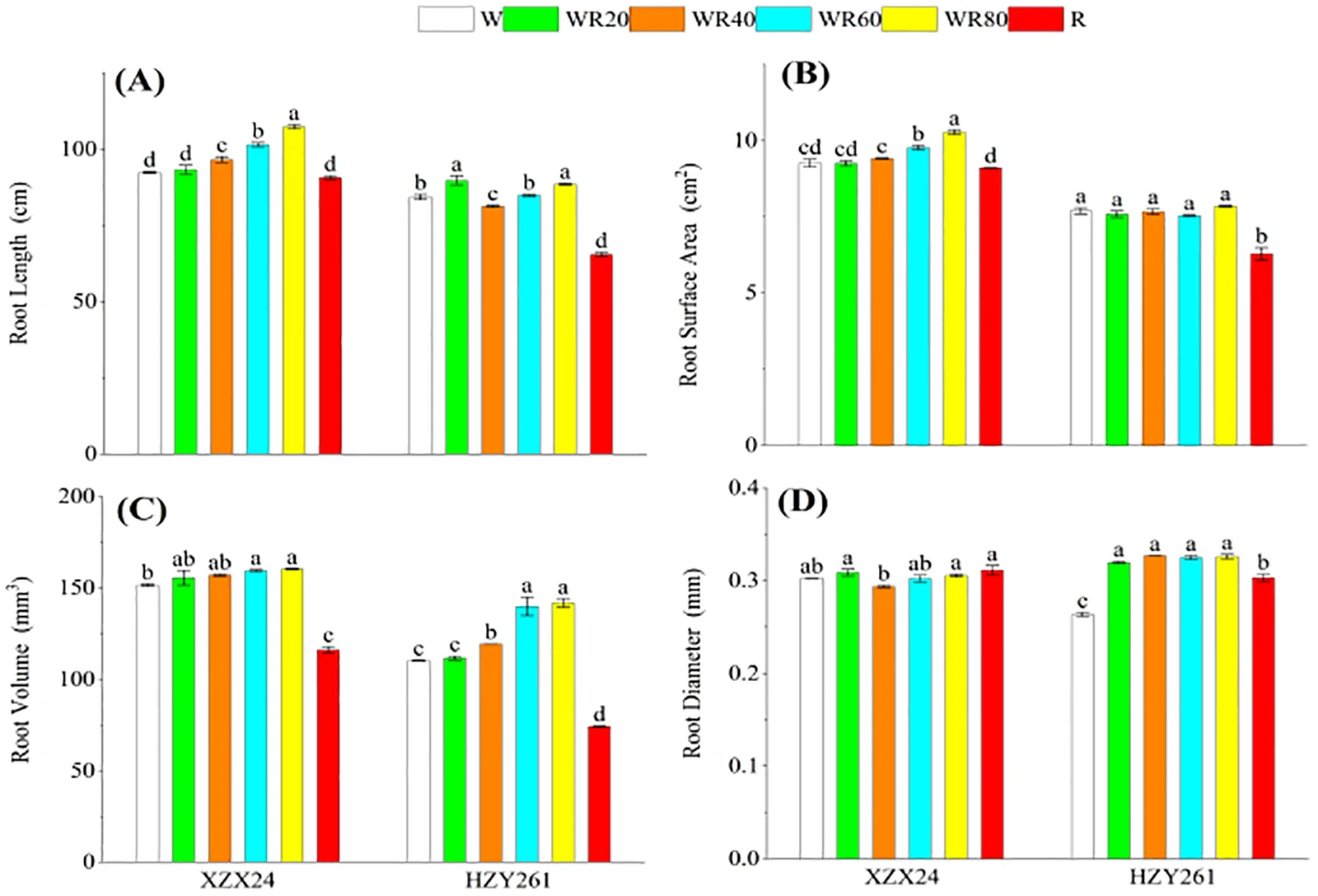
Impacts of varying red light treatment ratios on rice seedling root development: **(A)** Root length, **(B)** Root surface area, **(C)** Root volume, **(D)** Root diameter, XZX24: Xiangzaoxian24, HZY261: Huazheyou261, W: white light, WR20: 80% white light + 20% red light, WR40: 60% white light + 40% red light, WR60: 40% white light + 60% red light, WR80: 20% white light + 80% red light, R: 100% red light. Per the LSD test findings, distinct lowercase letters mark statistically significant contrasts among various light quality treatment conditions applied to the identical rice variety, the differences are statistically significant at the 5% significance level. The error bars above the average values represent the standard error (n = 18).

**Figure 4** illustrates how varying ratios of red light treatment impact the root growth of rice seedlings. The red light ratio significantly influences the root development of rice seedlings. Relative to the W treatment, the WR80 treatment results in a 10.97% (2.02% for HZY261) increase in root surface area and a 5.82% (28.62% for HZY261) rise in root volume for XZX24 seedlings. Meanwhile, in comparison to those treated with the W condition, the measurements of root length, root surface area, and root volume of HZY261 seedlings under the R treatment decrease by 22.42%, 18.32% and 32.74% (**Figures 4A–C**), respectively, while the root diameter of seedlings increases by 15.08% (**Figure 4D**).

### 3.4 Fresh weight, dry weight

**Figure 5:**
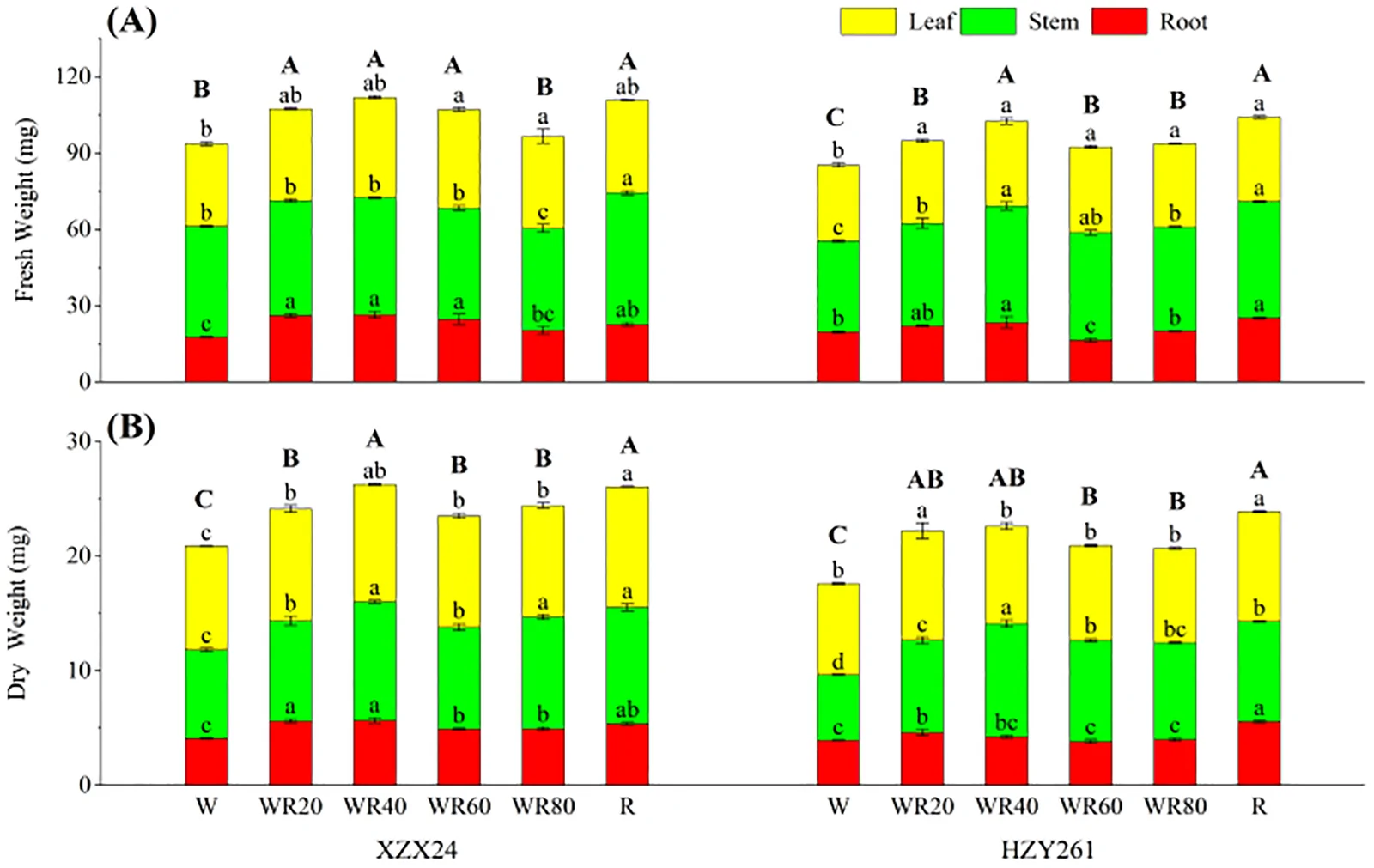
Effects of different proportions of red light treatment on the fresh weight and dry weight of rice seedlings. **(A)** Fresh weight, **(B)** Dry weight, XZX24: Xiangzaoxian24, HZY261: Huazheyou261, W: white light, WR20: 80% white light + 20% red light, WR40: 60% white light + 40% red light, WR60: 40% white light + 60% red light, WR80: 20% white light + 80% red light, R: 100% red light. Distinct lowercase (uppercase) letters denote statistically significant variations in root weight, stem weight, and leaf weight (or total weight) across treatments for the same rice variety. This difference is statistically significant at the 5% significance level, in accordance with the LSD test. The error bars above the average values represent the standard error (n = 60).

**Figure 5** displays the influence of varying red light treatment ratios on the dry and fresh weights of rice seedlings. In the white light + red light (WR20, WR40, WR60, WR80) treatments, the total fresh weight of rice seedlings increased first and then decreased as the proportion of red light increased. Compared with the W treatment, the total fresh weight of XZX24 (HZY261) seedlings in the WR20, WR40, WR60, WR80 treatment increased by 14.65%, 19.42%, 14.38%, 3.19% (11.35%, 20.33%, 8.43%, 9.97%). At the same time, compared with the W treatment, the total fresh weight of XZX24 (HZY261) seedlings in the R treatment increased by 18.31% (22.17%) (**Figure 5A**). Under white light plus red light (WR20, WR40, WR60, WR80) irradiation, the total dry biomass of rice seedlings initially increased, then decreased, and reached the maximum value as the red light proportion increased. Compared with the W treatment, the total dry weight of XZX24 (HZY261) seedlings in the WR20, WR40, WR60, WR80 treatment increased by 15.53%, 25.75%, 12.59%, 16.92% (26.19%, 28.56%, 18.88%, 17.55%). At the same time, compared with the W treatment, the total dry weight of XZX24 (HZY261) seedlings in the R treatment increased by 24.84% (35.72%) (**Figure 5B**).

### 3.5 Pigment content

**Figure 6:**
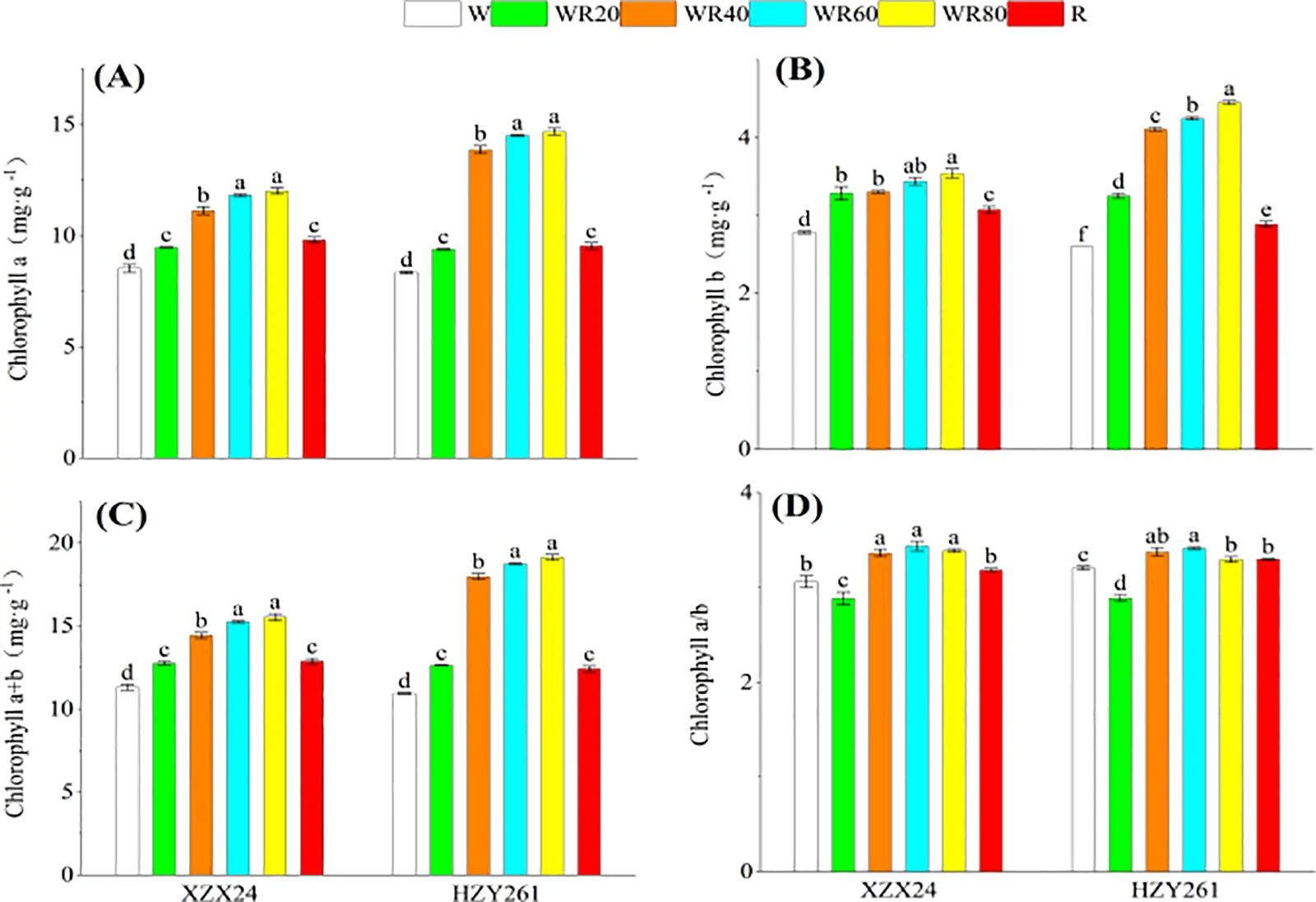
The impact of multiple fractions of red light treatment on pigment content of rice seedlings. **(A)** Chlorophyll a, **(B)** Chlorophyll b, **(C)** Chlorophyll a+b, **(D)** Chlorophyll a/b, XZX24: Xiangzaoxian24, HZY261: Huazheyou261, W: white light, WR20: 80% white light + 20% red light, WR40: 60% white light + 40% red light, WR60: 40% white light + 60% red light, WR80: 20% white light + 80% red light, R: 100% red light. Based on the LSD test findings, distinct lowercase letters denote significant differences among various light quality treatments applied to the same cultivar, the differences are statistically significant at the 5% significance level. The error bars above the average values represent the standard error (n = 18).

**Figure 6** illustrates how varying red light ratios impact the photosynthetic pigments in rice seedlings. In the white light + red light treatment (WR20, WR40, WR60, WR80), the chlorophyll a, chlorophyll b, and chlorophyll a+b in the rice seedlings showed an increasing trend as the proportion of red light increased. Compared with the control group W treatment, the WR80 treatment increased chlorophyll a in XZX24 (HZY261) seedlings by 40.84% (76.02%) (**Figure 6A**), chlorophyll b by 27.28% (71.26%) (**Figure 6B**), and the level of chlorophyll a+b also increased by 37.50% (74.89%) (**Figure 6C**). Compared with the control group W treatment, the WR60 treatment increased the chlorophyll a/b ratio in XZX24 (HZY261) seedlings by 12.21% (6.49%) (**Figure 6D**). At the same time, compared with the W treatment, in the R treatment, the chlorophyll a, chlorophyll b, chlorophyll a+b, and chlorophyll a/b of XZX24 (HZY261) seedlings increased by 14.99%, 10.54%, 13.89%, and 4.03% (respectively 14.23%, 11.08%, 13.48%, and 2.83%) (**Figures 6A–D**).

### 3.6 Variable chlorophyll fluorescence

**Figure 7:**
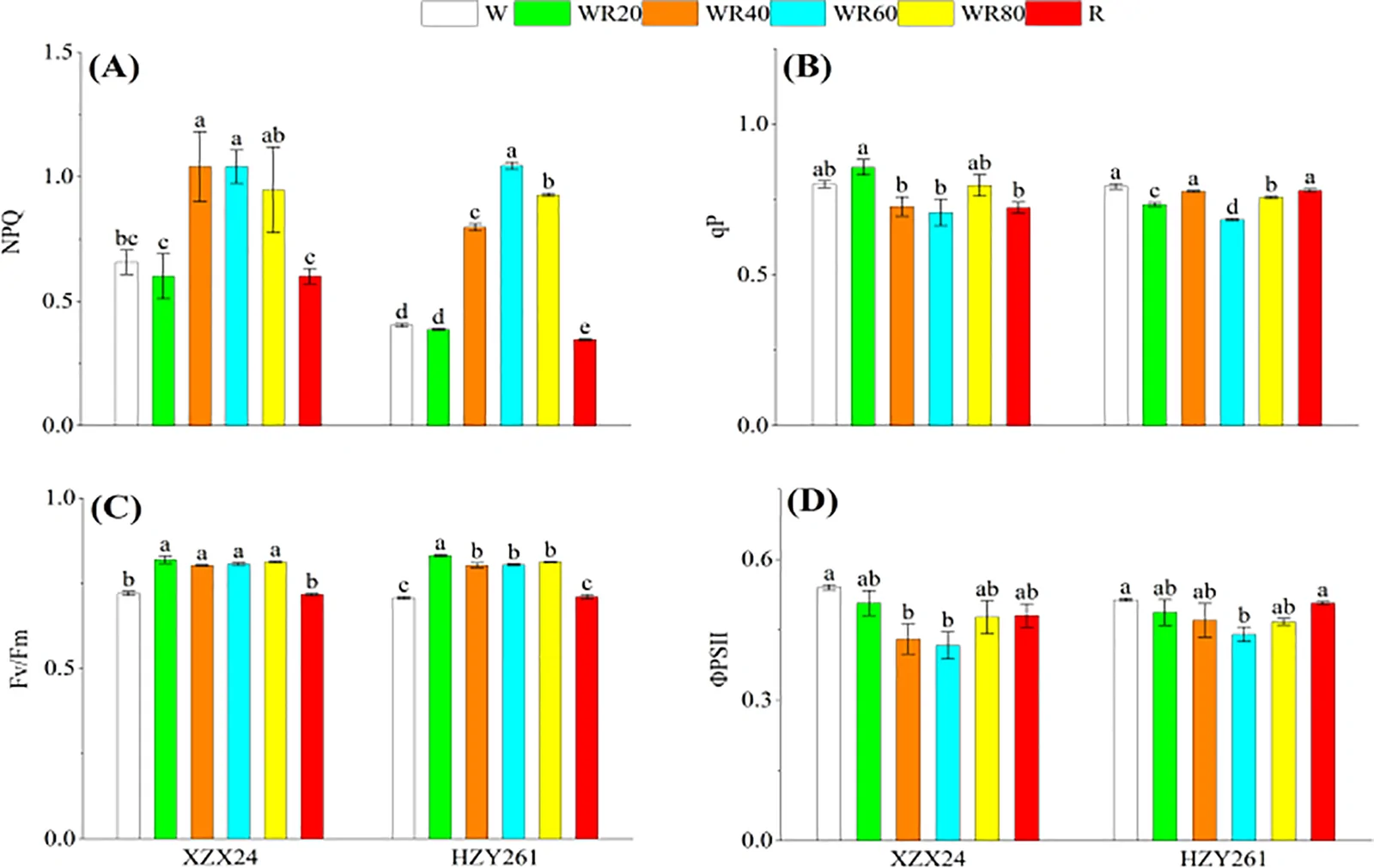
The impact of varying red light ratios with respect to the variable chlorophyll fluorescence in rice seedlings. **(A)** NPQ, **(B)** qP, **(C)** Fv/Fm, **(D)** ΦPSII, XZX24: Xiangzaoxian24, HZY261: Huazheyou261, W: white light, WR20: 80% white light + 20% red light, WR40: 60% white light + 40% red light, WR60: 40% white light + 60% red light, WR80: 20% white light + 80% red light, R: 100% red light. Consistent with the results of the LSD test, distinct small-case letters signify significant disparities among various light quality treatments applied to the identical variety, the differences are statistically significant at the 5% significance level. The error bars above the average values represent the standard error (n = 18).

Impacts of different red light treatment ratios on the variable chlorophyll fluorescence of rice seedlings are shown in **Figure 7**. In the white light + red light treatment (WR20, WR40, WR60, WR80), as the proportion of red light increased, the NPQ value in the leaves of rice seedlings first rose and then decreased. Compared with the control group (W), the NPQ values of XZX24 (HZY261) seedlings treated with WR40, WR60, and WR80 increased by 58.38%, 58.38%, and 44.16% (97.52%, 158.68%, 129.75%) (**Figure 7A**). In the white light + red light treatment (WR20, WR40, WR60, WR80), the qP values of XZX24 seedlings (HZY261) treated with WR40 and WR60 were significantly lower than W, while the qP values of HZY261 seedlings treated with WR20, WR60, and WR80 were significantly lower than W (**Figure 7B**). The Fv/Fm values of rice seedlings treated with white light + red light (WR20, WR40, WR60, WR80) were significantly higher than W (**Figure 7C**). Compared with the control group (W), the ΦPSII of XZX24 seedlings treated with WR40 and WR60 was significantly lower than W, and the ΦPSII of HZY261 seedlings treated with WR60 was significantly lower than W (**Figure 7D**). At the same time, compared with W, the NPQ, qP, and ΦPSII of XZX24 (HZY261) seedlings treated with R decreased by 8.63%, 9.58%, and 11.11% (14.05%, 1.68%, 0.47%, and 1.30%, respectively).

### 3.7 Photosynthesis

**Figure 8:**
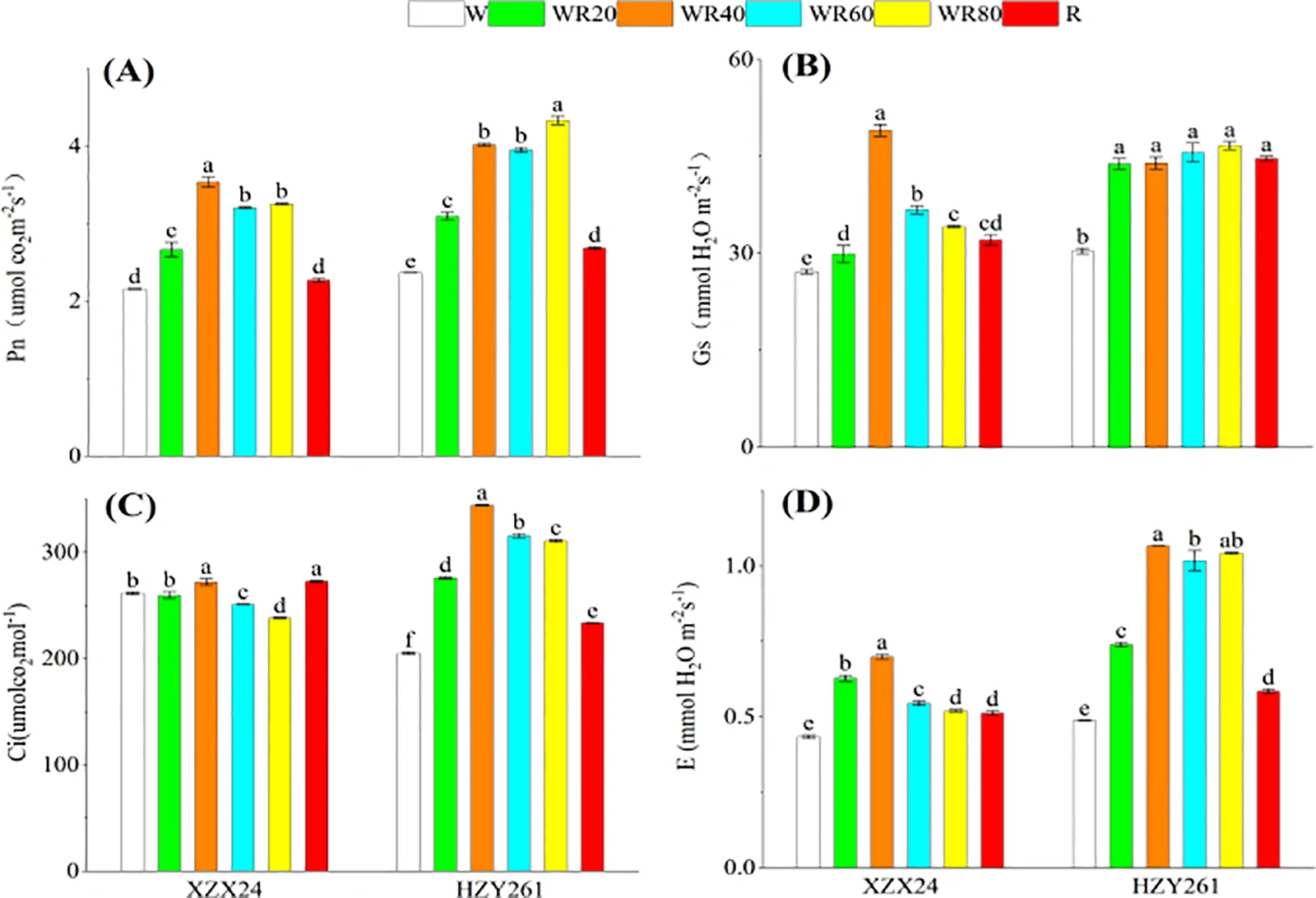
Influence of distinct red light treatment ratios on photosynthesis of rice seedlings. **(A)** Pn, **(B)** Gs, **(C)** Ci, **(D)** E, XZX24: Xiangzaoxian24, HZY261: Huazheyou261, W: white light, WR20: 80% white light + 20% red light, WR40: 60% white light + 40% red light, WR60: 40% white light + 60% red light, WR80: 20% white light + 80% red light, R: 100% red light. In light of the results of the LSD test, lowercase letters that differ denote notable variations among distinct light quality treatments for the same plant variety, the differences are statistically significant at the 5% significance level. The error bars above the average values represent the standard error (n = 18).

Impacts of different red light ratios regarding the photosynthetic attributes of rice seedlings are shown in **Figure 8**. Under the white light + red light treatment (WR20, WR40, WR60, WR80), the Pn, Gs and E values of the XZX24 seedlings reached their peak at WR40 (**Figures 8A, B, D**). Compared with the control group (W), the Pn, Gs and E of the XZX24 seedlings increased by 64.10%, 80.64% and 61.18% respectively (**Figures 8A, B, D**). Under the white light + red light treatment (WR20, WR40, WR60, WR80), the Pn of the HZY261 seedlings reached its maximum at WR80 (**Figure 8A**), while the Ci reached its peak at WR40 (**Figure 8C**). Meanwhile, compared with the control group, under the R treatment, the Pn, Gs, Ci and E of the XZX24 (HZY261) seedlings increased by 5.29%, 18.19%, 4.37% and 18.16% (13.69%, 47.34%, 14.06%, 19.68%) (**Figures 8A–D**).

### 3.8 Rubisco activity, gene expression

**Figure 9:**
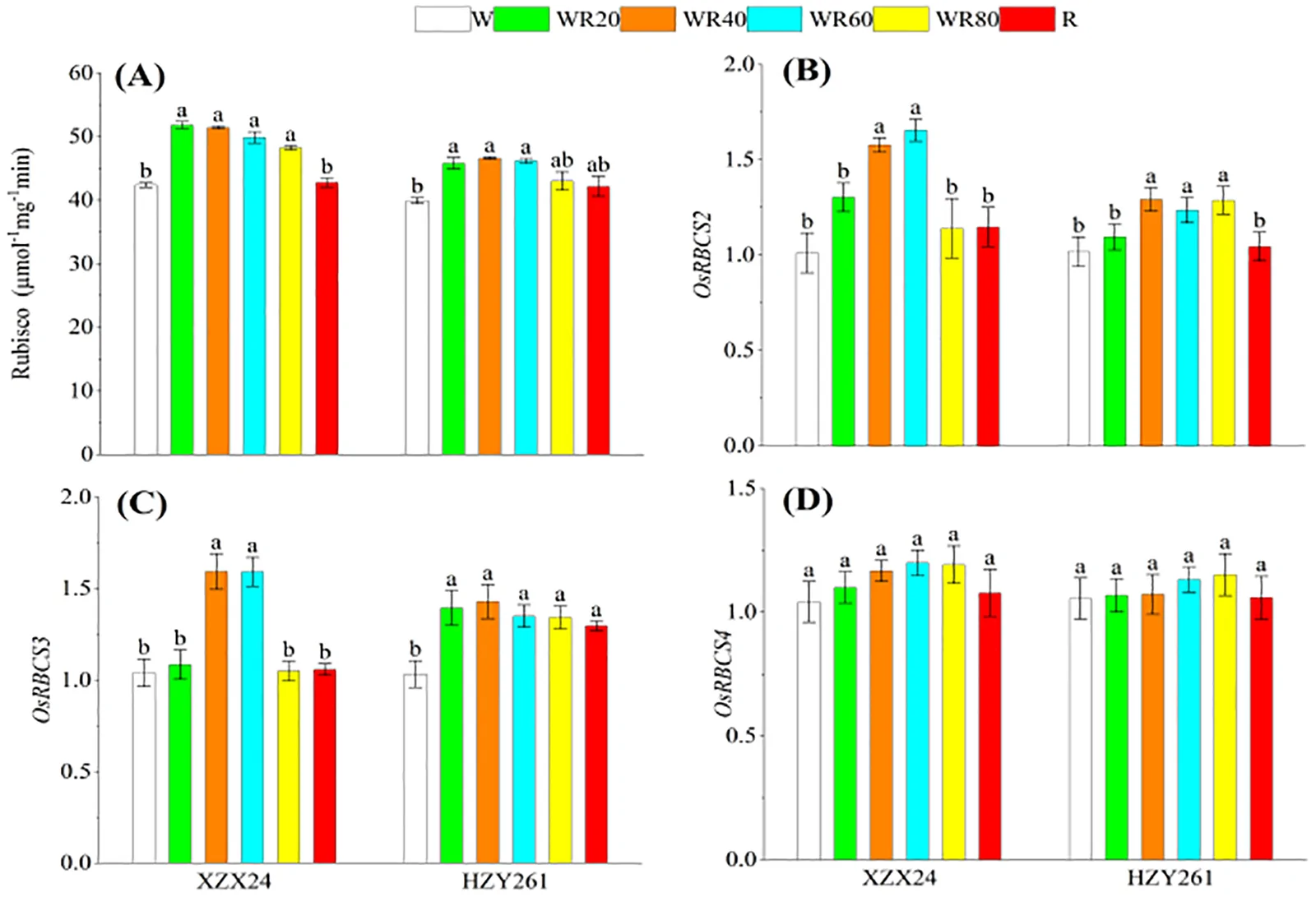
Effects of different proportions of red light treatment on the Rubisco activity, gene expression of rice seedlings. **(A)** Rubisco activity, **(B)** Relative expression level of *OsRBCS2*, **(C)** Relative expression level of *OsRBCS3*, **(D)** Relative expression level of *OsRBCS4*. Based on the LSD test findings, distinct lowercase letters denote significant differences among various light quality treatments applied to the same cultivar, the differences are statistically significant at the 5% significance level. The error bars above the average values represent the standard error (n = 3).

**Figure 9A** shows the Rubisco enzyme activity of XZX24 and HZY261. There are differences in Rubisco activity among varieties and among light quality treatments. The Rubisco enzyme activity of XZX24 rice seedlings under white light + red light treatment (WR20, WR40, WR60, WR80) was higher than that of W and R. Compared with the control group (W), the treatments of WR20, WR40, WR60 and WR80 increased the activity by 22.22%, 21.30%, 17.65% and 13.78% respectively. Under the white light + red light treatment (WR20, WR40, WR60), the Rubisco enzyme activity of HZY261 rice seedlings was higher than that of W. Compared with the control group, the treatments of WR20, WR40, WR60 increased the activity by 14.72%, 16.75%, and 15.62% respectively. Meanwhile, compared with the control group, under R treatment, the Rubisco enzyme activity of XZX24 (HZY261) rice seedlings increased by 0.90% (5.89%).

Different light qualities affect the expression levels of the photosynthetic genes *OsRBCS2*, *OsRBCS3*, and *OsRBCS4* in rice leaves (**Figures 9B–D**). The expression level of *OsRBCS2* in XZX24 rice seedlings was significantly higher in WR40 and WR60 compared to other treatments, and compared to W, WR40 and WR60 treatments increased by 55.99% and 63.71%, respectively; for HZY261 rice seedlings, the expression level of *OsRBCS2* was significantly higher in WR40, WR60, and WR80 compared to other treatments, and compared to W, WR40, WR60, and WR80 treatments increased by 26.97%, 21.46%, and 26.59%, respectively (**Figure 9B**). In the XZX24 rice seedlings, the expression levels of *OsRBCS3* in the WR40 and WR60 treatments were significantly higher than those in the other treatments. The expression levels in the WR40 and WR60 treatments were 53.09% and 53.02% higher than that in the W treatment, respectively. For the HZY261 rice seedlings, the expression levels of *OsRBCS3* in all treatments were significantly higher than that in the W treatment. The expression levels in W, WR20, WR40, WR60, WR80, and R treatments were 34.96%, 38.31%, 30.68%, 29.91%, and 25.45% higher than that in the W treatment, respectively (**Figure 9C**). In addition, the study also found that although different light qualities have an impact on the expression level of *OsRBCS4*, the differences among the treatments are not significant (**Figure 9D**).

### 3.9 Heat map analysis

**Figure 10:**
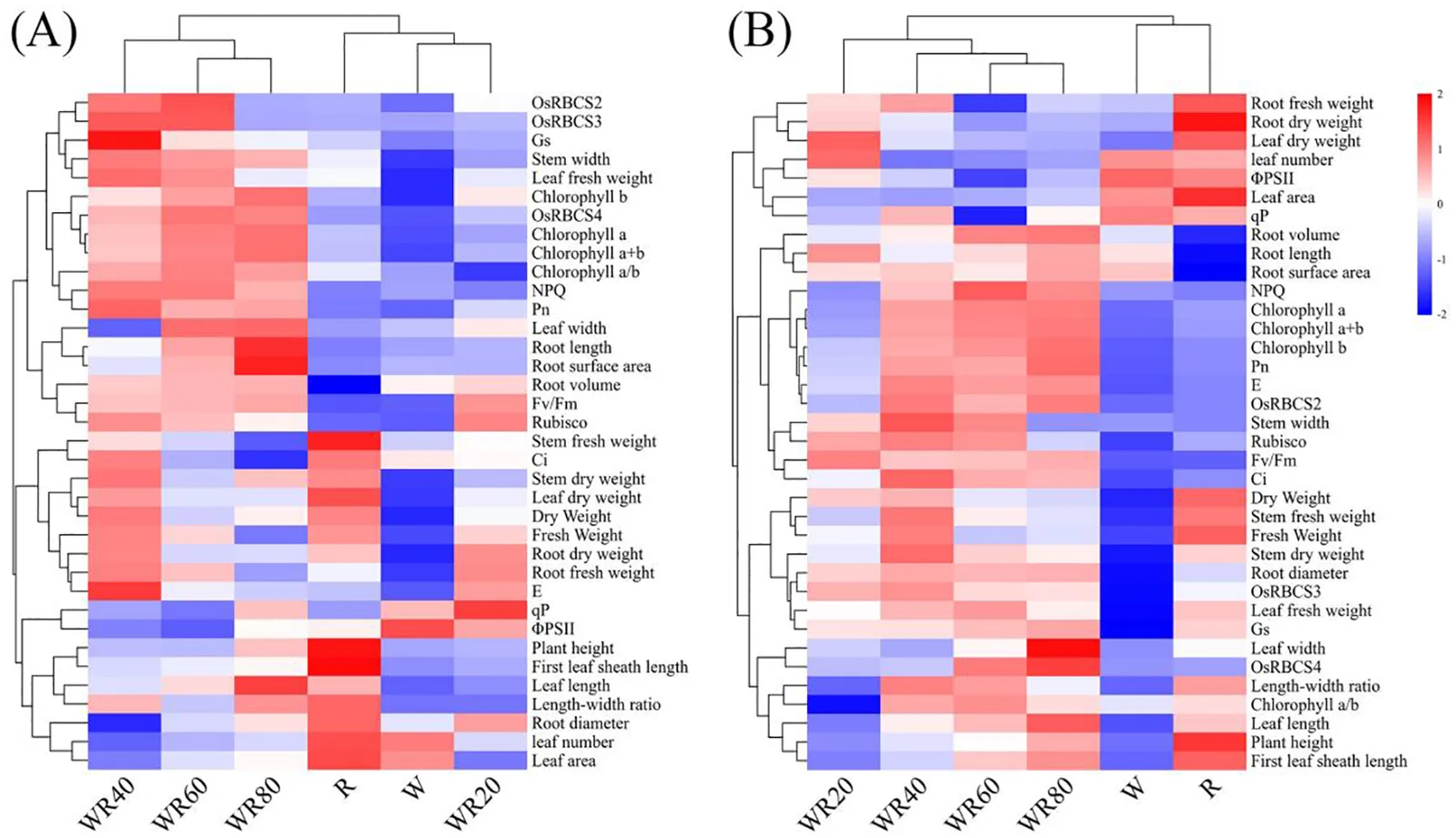
Responses of rice seedlings to different proportions of red light treatment through hierarchical clustering heatmap analysis. **(A)** XZX24, **(B)** HZY261, W: white light, WR20: 80% white light + 20% red light, WR40: 60% white light + 40% red light, WR60: 40% white light + 60% red light, WR80: 20% white light + 80% red light, R: 100% red light. These results are presented through a pseudo-color plot, where red indicates a rise in the response parameter, while blue represents a decline in it.

Within the XZX24 group, the W and WR20 clusters showed the most similar responses to the measurement parameters, while the WR60 and WR80 clusters also had relatively similar responses (**Figure 10A**). Additionally, the distance between the W and WR20 clusters was the same as that between the R cluster, and the distance between the WR60 and WR80 clusters was the same as that between the WR40 cluster. Meanwhile, the WR40 cluster was clearly separated from the WR60 and WR80 clusters: the light of WR40 reduced leaf width, qP, ΦPSII, root diameter, leaf number, leaf area, and increased *OsRBCS2*, *OsRBCS3*, Gs, stem width, leaf fresh weight, NPQ, Pn, Rubisco, Ci, stem fresh weight, dry weight, E. The R cluster also clearly separated from the W and WR20 clusters: the light of R reduced *OsRBCS2*, *OsRBCS3*, *OsRBCS4*, chlorophyll b, NPQ, Pn, leaf width, root length, root surface area, root volume, Fv/Fm, Rubisco, qP, and increased stem fresh weight, Ci, stem dry weight, leaf dry weight, dry weight, fresh weight, root fresh weight, plant height, first leaf sheath length, leaf length, length-width ratio, root diameter, leaf number, leaf area, were instrumental in differentiating the R cluster from the remaining clusters.

Among the HZY261, the WR60 and WR80 clusters showed the most similar responses to the measurement parameters, while the W and R clusters also had relatively similar responses (**Figure 10B**). Additionally, the distances between WR40 and WR60, WR80 were the same. The WR20 cluster also clearly separated from the WR40, WR60, WR80 and W, R clusters: the light of WR20 reduced leaf area, qP, NPQ, chlorophyll a, chlorophyll a+b, *OsRBCS2*, *OsRBCS4*, stem fresh weight, length-width ratio, chlorophyll a/b, leaf length, plant height, first leaf sheath length, and increased leaf fresh weight, leaf number, root length, Rubisco, Fv/Fm, were instrumental in differentiating the WR20 cluster from the remaining clusters.

## 4 Discussion

### 4.1 Red light regulates the morphological development and biomass accumulation of rice seedlings

Studies have shown that adding red light to white light for cultivating rice seedlings has a regulatory effect on the morphological formation and biomass accumulation of rice seedlings (**Figures 2**-**5**) (Wang et al., 2024). The height of rice seedlings increases with the increase in the proportion of red light, reaching the maximum value under pure single red light (R) conditions (**Figure 2A**) (Wei et al., 2023b). It has been shown that red light significantly promotes the longitudinal elongation of the aboveground tissues of rice seedlings (Rahman et al., 2024). In contrast, stem width shows a unimodal curve, and the value of stem diameter in the WR40 (60% white light + 40% red light) treatment is the largest (**Figure 2C**) (Heidari et al., 2023). This indicates that an appropriate amount of red light supplementation can coordinate the vertical elongation at the stem base with lateral cell division, while excessive single red light will interfere with lateral development, resulting in poor growth of the seedlings (Zhang et al., 2022; Dutta Gupta and Jatothu, 2013).

It was found that a higher proportion of red light has a significant effect on the increase in leaf area of rice seedlings (**Figure 3**), but pure red light has a significant inhibitory effect on root length, root surface area, and root volume (**Figure 4**). Under the R treatment, the root volume of XZX24 decreased by 23.29%, HZY261 by 32.74%, while the root diameter only slightly increased. This shows that a single red light can only make the single root canopy structure thicker, but cannot promote the branching and expansion of the entire root system (Ikeura et al., 2026). The total fresh weight and dry weight of all white-red light combined treatments were significantly higher than the W treatment, and the dry-to-wet weight ratio of rice seedlings reached the maximum at WR40 (**Figure 5**), where the total dry weight of the two rice varieties increased by more than 20%. The hierarchical clustering heatmap results (**Figure 10**) show that WR40 formed an independent phenotypic group, characterized by robust stems, a balanced root system structure, and the highest dry matter accumulation, which is more in line with the agronomic standards of healthy rice seedlings (Wei et al., 2020). Although red light treatment simultaneously increased plant height and leaf area, its defective root system disrupted the coordinated growth balance between the stem and the root.

Red light can be sensed by the photoreceptors of rice and regulate the synthesis speed of related genes, thereby accelerating the longitudinal elongation of stem and leaf sheath cells (Xiao et al., 2025). This mechanism explains the phenomenon of continuous growth in plant height and leaf sheath length under the high red light ratio treatment (**Figures 2A, B**) (Bai et al., 2024). Most previous related studies only used pure monochromatic light or simple red-blue mixed light, did not use natural white light as the basic light source, and only concluded that red light can promote the elongation of seedlings, ignoring its inhibitory effect on the lateral development of stems and roots. In this study, this inhibitory effect was manifested in stem width and root morphology parameters (**Figures 2C**, **4**) (Seung and Kweon, 2023; Zhou et al., 2025). The gradient white-red light spectrum system constructed in this experiment further clarified the balancing function of white light components: the synergy effect of white light helps maintain the cell division activity of the apical meristem of the root tip and effectively alleviates the growth retardation of the root system caused by single red light stress (V. Kurepin et al., 2007; Srinidhi and Scott, 2017).

Under the WR40 light condition, the synchronous maximum values of stem width and total dry matter revealed the key intrinsic coupling relationship between the lateral growth of rice seedlings and the accumulation of organic matter. The relatively thick stem base can improve the efficiency of transporting photosynthetic products synthesized by leaves to the underground root system, while the developed root system promotes the absorption of water and mineral nutrients, thereby maintaining the continuous expansion of leaves and forming a positive feedback loop between aboveground and underground organs. Under pure red light irradiation, the mutual restraint between vigorous aboveground growth and poor root development indicates that the higher the red light ratio, the quality of the seedlings may not necessarily be better (Lawton and Magnusson, 2025; Johnson et al., 2020). Therefore, this study provides a multi-dimensional morphological basis for achieving factory-based, high-yield, and high-quality seedling cultivation. In addition, WR40 showed continuous and stable optimal performance in stem thickness and biomass in both rice varieties, providing a universal reference spectrum for early rice seedling cultivation.

The limitations of this study and future research prospects it should be noted that all the morphological response patterns summarized in this study were obtained under stable and constant temperature and humidity conditions in an artificial climate chamber. The selected light spectrum scheme here cannot be directly replicated and applied to the actual production of rice seedlings in facilities, because the temperature, light duration, and humidity in the natural environment fluctuate. In actual protective seedling sheds, factors such as continuous rainy days, diurnal temperature differences, and different densities of seedling trays will jointly affect the regulatory effect of red-white composite light on the morphology and root growth of rice seedlings. Therefore, the optimal spectral matching standard proposed in this paper still needs to be verified in the field through targeted experiments in greenhouse seedling beds.

### 4.2 Regulation of photosynthetic pigments, chlorophyll fluorescence, and carbon fixation capacity by the white-red composite spectrum

By comprehensively analyzing the data of leaf pigments, chlorophyll fluorescence, gas exchange, Rubisco activity and gene expression, a complete response chain of the rice photosynthetic apparatus to the gradient of white and red light spectra was constructed, covering the processes of light energy capture, photochemical conversion, photoprotection and carbon fixation (**Figures 6**-**9**). In the four mixed white and red light treatments, as the proportion of red light increased, the contents of chlorophyll a, chlorophyll b and total chlorophyll gradually rose (**Figure 6**) (Lopez-Figueroa, 1991; Xin et al., 2026). Compared with the white light control group (W), the increase in red light promoted the formation of chlorophyll, and monochromatic red light (R) cultivation was not conducive to the formation of photosynthetic pigments. At the same time, studies have shown that the ratio of chlorophyll a/b also changes with the variation of the proportion of red light (Bi et al., 2024), indicating that a moderate supplementation of red light can optimize the proportion balance between the primary reaction center chlorophyll a and the auxiliary photosynthetic pigment chlorophyll b, thereby improving the absorption efficiency of red light by the leaf tissue.

The intensity changes of chlorophyll fluorescence can directly reflect the state of a plant’s photosynthetic efficiency (**Figure 7**) (Chen et al., 2023). The study found that in the same light intensity environment, compared with white light and single-color red light treatments, adding red light treatments (WR20, WR40, WR60, WR80) resulted in higher maximum photochemical efficiency Fv/Fm in rice seedlings (**Figure 7C**). This indicates that supplementing red light in the background of white light can effectively improve the efficiency of converting captured light energy into chemical reduction capacity. Non-photochemical quenching (NPQ) is a key indicator for dissipating excess light energy and reducing light damage (Saber et al., 2018), and the study found that the NPQ of rice seedlings cultivated with a certain proportion of red light added on the basis of white light was higher (**Figure 7A**). In contrast, pure red light significantly reduced NPQ, qP, and ΦPSII simultaneously, weakening the light protection ability of the seedling leaves under changing light conditions.

The study found that in the same light intensity environment, compared with white light and monochromatic red light treatments, the net photosynthetic rate of rice seedlings treated with red light addition (WR20, WR40, WR60, WR80) was higher (**Figure 7C**). This indicates that supplementing red light in the white light background can effectively improve the net photosynthetic efficiency of plant leaves. Under the medium red light addition treatment (WR20–WR60), the Rubisco carboxylation activity and the transcriptional abundance of *OsRBCS2* and *OsRBCS3* remained stable (Halimeh, 2025; Karagülle and Telli, 2024). Monochromatic red light (R) only slightly increased Rubisco activity and failed to induce high-level expression of these two key Rubisco subunit genes. The combined analysis of all photosynthetic indicators revealed that there are differences in the optimal spectra of different functional layers of the photosynthetic system: WR80 is most conducive to the accumulation of photosynthetic pigments, WR60 optimizes the light protection ability by increasing NPQ, and WR40 achieves a balance between Rubisco activity and the transcription of carbon assimilation genes, thereby achieving a higher overall carbon assimilation efficiency. These multi-index differences directly explain why WR40 produced the highest dry matter of seedlings in morphological measurements (**Figure 5**).

Chlorophyll a and b possess major absorption bands within the red wavelength range (Han et al., 2017; Wang et al., 2021). Consistent with previous pigment research, our gradient red light treatments triggered continuous pigment accumulation in rice leaves by promoting the assembly of thylakoid pigment-protein complexes (Ashkovskiy et al., 2023). Pigment promotion was far weaker under pure red light than WR80, which can be attributed to the absence of full-spectrum white light components required to sustain steady pigment synthesis. Most prior light-quality evaluations rely solely on single indicators such as Pn or chlorophyll concentration, lacking joint analysis of pigments, fluorescence, gas exchange, Rubisco activity and carbon fixation gene expression.

The six gradient white-red light treatments in this study enabled systematic linkage of multiple photosynthetic indices to establish a complete carbon assimilation response chain (Di et al., 2023; Dewir et al., 2023). Transcription of *OsRBCS2* and *OsRBCS3* showed clear positive responses to appropriate mixed white-red spectra, whereas no significant treatment-induced variation was detected for *OsRBCS4* (**Figure 9**) (Nie et al., 2024). Our dataset proves maximum pigment concentration (WR80) cannot be equated to superior photoprotection (WR60) or peak carbon fixation efficiency (WR40 for XZX24; WR80 for HZY261). Relying merely on chlorophyll content as a screening standard for supplementary light will overlook differences in light stress tolerance and dry matter production potential (Wei et al., 2023a; Zhang et al., 2024a). Collectively, the multi-dimensional quantitative photosynthetic data generated in this work provides differentiated spectral evaluation criteria for intelligent supplementary lighting, and clarifies the coordinated physiological responses of rice seedling photosynthetic apparatus to mixed white-red spectra, based entirely on the measured physiological and molecular indicators obtained in our experiment.

## 5 Conclusions

Graded supplementary red light exerted divergent regulatory effects on the growth, photosynthetic performance and Rubisco-related gene expression of seedlings of two rice cultivars. Among all tested treatments, the WR40 regime facilitated better seedling morphological development, higher photosynthetic pigment concentration and enhanced Rubisco activity. This spectral combination can be adopted as a preferred light environment reference for indoor factory rice seedling raising. Variations in red light proportion modified leaf photochemical efficiency and biomass accumulation. Excessively high fractions of monochromatic red light (R) failed to sustain continuous improvement in seedling quality, indicating that appropriate white–red light matching is essential for cultivating robust rice seedlings. Light quality regulation altered the transcript abundance of Rubisco coding genes. Treatments with increasing red light ratios significantly regulated the expression of *OsRBCS2* and *OsRBCS3*, whereas no significant treatment effect was detected for *OsRBCS4*. The transcriptional responses of these two genes partially explain the changes in leaf photosynthetic capacity under different light spectra. Although both rice genotypes displayed generally consistent response trends to red light gradients, further verification under variable environmental conditions is still required. Future research should investigate the interactive effects of light quality with temperature, water and nutrient supply, and validate the practicability of the WR40 spectral scheme in large-scale commercial seedling workshops.

## Data Availability

The original contributions presented in the study are included in the article/**Supplementary Material**. Further inquiries can be directed to the corresponding author.
