# Supplementary material for: Red light regulation on growth, root-leaf architecture and photosynthetic performance of rice seedlings
Source: Front Plant Sci. 2026 Sep 9;17:1919485. doi: 10.3389/fpls.2026.1919485 (PMC13597676; doi:10.3389/fpls.2026.1919485)
Supplement: Supplementary file 1 [file Table1.docx]

Supplementary Table 1: Two-factor Analysis of Variance for Relevant Indicators

|  | Plant Height | | Leaf Area | | Chlorophyll a+b | | Pn | | Total Dry Weight | |
| --- | --- | --- | --- | --- | --- | --- | --- | --- | --- | --- |
|  | F | *P* | F | *P* | F | *P* | F | *P* | F | *P* |
| Variety | 63.273 | <0.001 | 754.051 | <0.001 | 366.024 | <0.001 | 555.436 | <0.001 | 97.848 | <0.001 |
| Treatment | 243.351 | <0.001 | 184.135 | <0.001 | 659.710 | <0.001 | 544.235 | <0.001 | 32.036 | <0.001 |
| Variet×Treatment | 9.782 | <0.001 | 14.453 | <0.001 | 104.914 | <0.001 | 27.904 | <0.001 | 1.144 | 0.365 |
